# An Overview of Diabetes Management in Schizophrenia Patients: Office Based Strategies for Primary Care Practitioners and Endocrinologists

**DOI:** 10.1155/2015/969182

**Published:** 2015-03-23

**Authors:** Aniyizhai Annamalai, Cenk Tek

**Affiliations:** ^1^Departments of Psychiatry and Internal Medicine, Yale School of Medicine, 34 Park Street, New Haven, CT 06519, USA; ^2^Department of Psychiatry, Yale School of Medicine, 34 Park Street, New Haven, CT 06519, USA

## Abstract

Diabetes is common and seen in one in five patients with schizophrenia. It is more prevalent than in the general population and contributes to the increased morbidity and shortened lifespan seen in this population. However, screening and treatment for diabetes and other metabolic conditions remain poor for these patients. Multiple factors including genetic risk, neurobiologic mechanisms, psychotropic medications, and environmental factors contribute to the increased prevalence of diabetes. Primary care physicians should be aware of adverse effects of psychotropic medications that can cause or exacerbate diabetes and its complications. Management of diabetes requires physicians to tailor treatment recommendations to address special needs of this population. In addition to behavioral interventions, medications such as metformin have shown promise in attenuating weight loss and preventing hyperglycemia in those patients being treated with antipsychotic medications. Targeted diabetes prevention and treatment is critical in patients with schizophrenia and evidence-based interventions should be considered early in the course of treatment. This paper reviews the prevalence, etiology, and treatment of diabetes in schizophrenia and outlines office based interventions for physicians treating this vulnerable population.

## 1. Introduction

People with schizophrenia have an increased risk of diabetes and other metabolic abnormalities. A renewed interest in this phenomenon has been sparked by the adverse metabolic effects of antipsychotic medications used in the treatment of schizophrenia. It is now well established that people with serious mental illness (SMI), including schizophrenia, have excess morbidity and mortality leading to a reduced lifespan of 20–25 years compared with the rest of the population [1, 2]. The increased mortality is largely attributable to physical illness, including metabolic abnormalities and cardiovascular disease, rather than factors that are directly associated with psychiatric illness such as suicide or homicide. Metabolic syndrome occurs in one in three patients and diabetes in one in five patients [3]. These abnormalities not only confer an elevated cardiovascular risk and increased mortality in those with schizophrenia and other mental illness [4], but also are associated with poor psychiatric and functional outcomes [5].

For many people with schizophrenia and other serious mental illness, the mental health center is the primary point of contact with the health care system [6]. But there are multiple barriers to adequate screening and treatment at the mental health centers [7]. Referrals to community medical providers are challenging, in part due to administrative barriers, lack of communication between mental health and primary care practitioners and clinics, and also poor patient experience in medical settings. For patients with schizophrenia, psychiatric symptoms and cognitive deficits limit their social functioning and a fast paced medical health care environment is difficult to navigate. In one survey, these patients cited continuity of care and listening skills as qualities important in medical practitioners [8, 9].

Patients with SMI are usually on treatments that include psychopharmacologic agents, psychotherapy, and other social interventions. Antipsychotics are a cornerstone of treatment in those with schizophrenia. A category of agents, known as second-generation antipsychotics, have been used since the early 1990s and in the last two decades there has been a tremendous increase in use of these medications [9]. These agents are now known to contribute significantly to obesity and metabolic syndrome, though there are variations in magnitude of risk between individual agents [10]. This, along with the increased smoking rates seen in people with schizophrenia, results in an increased cardiovascular risk and ultimately leads to worsened mortality rates [4, 11].

In spite of increased awareness among mental health providers of the increased prevalence of metabolic syndrome in SMI, rates of screening and treatment remain poor [12]. The mortality gap between this patient group and the rest of the population, largely due to medical illnesses, has not narrowed [13]. Primary care providers already have the specialized medical knowledge necessary to treat medical conditions in those with schizophrenia. Increased awareness among medical providers of the high medical morbidity and mortality in schizophrenia is critical. Skills of primary care such as empathic listening, targeted education, continuity of care, and care coordination with mental health providers all have the potential to significantly improve the health of patients with schizophrenia.

## 2. Prevalence of Diabetes Diagnosis and Treatment in Schizophrenia

The rate of metabolic syndrome and diabetes in patients with schizophrenia is higher than the general population. A meta-analysis of several studies comprising over 25,000 patients with schizophrenia and related disorders showed an overall rate of metabolic syndrome at 32.5% and hyperglycemia at 19% [3].

A large multisite study, the Clinical Antipsychotic Trials of Intervention Effectiveness (CATIE), examined the effectiveness of different antipsychotic medications in over 1400 patients with schizophrenia. In addition to psychiatric outcomes, the study also examined physical health indicators. Metabolic syndrome was seen in more than 40% of patients. Diabetes was seen in 11% of patients and fasting glucose levels >100 mg/dl were seen in more than 25% of patients in this study. Rates of treatment for metabolic syndrome were low with more than 30% of patients with diabetes not receiving treatment [12].

The prevalence of diabetes in schizophrenia has been estimated to be 2-3-fold higher than in the general population and estimates of prevalence range from 10 to 15% [14]. In a study of over 400 patients with schizophrenia, the prevalence of diabetes and impaired glucose tolerance was 6.3% and 23.4%, respectively, in the total sample [15]. Diabetes prevalence was 4-5 times higher within each age group. The difference in diabetes prevalence between those with schizophrenia and the general population rose linearly with age from 1.6% in the 15–25 age group to 19.2% in the 55–65 age group. Interestingly, while the prevalence of metabolic syndrome is higher in those with schizophrenia, the increase in prevalence with age is the same as the general population. This suggests that the development of diabetes in schizophrenia is not solely secondary to metabolic syndrome but there may also be an inherent vulnerability to diabetes, possibly aggravated by pharmacological effects of some antipsychotics [16].

A large number of patients with schizophrenia and other SMI receive their psychiatric care at specialized mental health settings. A national screening program of 10,084 patients over several of these centers showed 37% of patients with schizophrenia had an elevated fasting glucose (>100 mg/dl) [17]. The rates of treatment were low, even among those with known diabetes. Approximately 40% of patients with schizophrenia and diagnosed diabetes reported not receiving any antihyperglycemics. This corroborates with the low rates of treatment seen in the CATIE study.

## 3. Etiology of Development of Diabetes in Schizophrenia

A link between schizophrenia and diabetes has been known for over a century, long before the use of antipsychotic medications. There is debate about the degree of contribution of genetics and environmental factors to development of diabetes.

Epidemiological studies show an increased risk of developing diabetes in people with schizophrenia with and without antipsychotics [18]. Some studies of people with antipsychotic naïve, first episode schizophrenia show impaired glucose tolerance and higher insulin resistance compared to healthy cohorts [19].

There is also evidence that antipsychotics increase metabolic risks, with second-generation agents showing differentially higher risk over time compared to first generation agents [20]. A recent comparative meta-analysis of metabolic abnormalities among unmedicated and first episode patients with schizophrenia showed a comparable rate with the general population [21]. These rates were much lower when compared with people with chronic schizophrenia established on medications. This would imply that most, if not all, the metabolic risk in schizophrenia patients is conferred by antipsychotic agents.

As can be seen, the data on the extent to which antipsychotics confer metabolic risk are conflicting. The relative contributions of genetic susceptibility and antipsychotic treatment to increased prevalence of diabetes are uncertain. The following sections review briefly some mechanisms postulated to explain the association of diabetes with schizophrenia.

### 3.1. Genetic Susceptibility to Diabetes

A common inherited susceptibility to both diabetes and schizophrenia has been postulated based on the observation that diabetes is more common in family members of those with schizophrenia [22]. A common genetic linkage between the two diseases has been suggested. Some authors report abnormal glucose metabolism and insulin signaling in the brain of those with schizophrenia [19, 23]. Genes involved in both glucose metabolism and cognitive function may increase the risk of diabetes in schizophrenia patients. There is also a suggestion of a common molecular mechanism underlying both cognitive deficits such as working memory and glucose metabolism [23].

### 3.2. Neuroendocrine Pathways Increasing Diabetes Risk

Some studies have reported dysregulation of the hypothalamic pituitary axis and high serum cortisol levels in people with schizophrenia. Elevated serum cortisol increases gluconeogenesis, insulin resistance, and symptoms of metabolic syndrome. It is hypothesized that the elevated cortisol also increases serum leptin levels with a resultant increase in appetite [24]. Some authors have also implicated nutritional factors in a common pathway for development of both diabetes and schizophrenia [25]. For example, a low level of vitamin D during childhood may be associated with schizophrenia and vitamin D may affect insulin response to glucose stimulation [26]. Gestational zinc deficiency has also been proposed as a possible mediator of a common etiologic pathway [27].

### 3.3. Antipsychotics and Risk of Diabetes

Antipsychotics lead to weight gain and a higher risk of obesity related complications including diabetes [20]. The metabolic effects on glucose and insulin metabolism between agents within each class of antipsychotics are different [28]. Second-generation or atypical antipsychotics had three times the rate of new onset metabolic syndrome compared to first generation or typical or conventional antipsychotics after three years on medications [20]. At the three-year follow-up, impaired fasting glucose was more frequent in those treated with the second-generation agents. But the difference between the two groups of agents was not significant when clozapine and olanzapine were excluded from the analysis. Both clozapine and olanzapine are second-generation antipsychotics.

In a large meta-analysis comprising 25,992 patients, one in five patients with schizophrenia had hyperglycemia; the rate of metabolic syndrome was 51.9% for clozapine, 28.2% for olanzapine, and 27.9% for risperidone [3]. Risperidone is also a second-generation agent.

These medications cause weight gain by multiple mechanisms mediated by their effect on hypothalamic regulation and action on dopaminergic, serotoninergic, and histaminergic receptors [29]. The resulting obesity is a risk for hyperglycemia but antipsychotics can also directly cause diabetes. One postulated mechanism is the ability of antipsychotics to block the pancreatic muscarinic (M3) receptor. Leptin resistance is another proposed mechanism.

### 3.4. Nonmedication Environmental Factors

It is well known that patients with schizophrenia and other serious mental illnesses have unhealthy lifestyles with poor diets and inadequate physical activity [30–33]. This places them at higher risk of obesity and other metabolic complications. Factors that contribute to poor access to healthy lifestyle choices in individuals with schizophrenia are lower socioeconomic status, lower educational level, living situation (residential settings and living in areas with abundance of fast food facilities), and social isolation. Symptoms of schizophrenia such as low motivation, apathy, and cognitive deficits also could play a role in preventing access to healthy lifestyles. Patients with schizophrenia are also much more likely to be dependent on tobacco [34] and this further increases risk for cardiovascular disease. The following is a summary of the proposed common pathways between the two disease conditions. 

*Relationship between Diabetes and Schizophrenia*

- Genetic susceptibility:
  - higher occurrence of diabetes in family members of schizophrenia patients [22],
  - abnormal glucose metabolism in schizophrenia patients [19, 23],
  - common mechanism proposed for cognitive deficit and glucose metabolism [23].
- Neuroendocrine pathways:
  - hypothalamic axis dysregulation and elevated cortisol in schizophrenia [24],
  - nutritional deficiencies proposed as common pathway for both diseases [25–27].
- Antipsychotic medications:
  - effect on hypothalamic regulation, dopaminergic, serotonergic, and histaminergic receptors [29],
  - other proposed mechanisms: action on pancreatic muscarinic receptor and leptin resistance [29].
- Environmental:
  - poor diet and lack of access to quality foods [30–32],
  - inadequate physical activity due to symptoms and social isolation [30, 31, 33].

## 4. Treatment of Diabetes in Schizophrenia

Developing effective treatment programs for diabetes care is imperative for people with schizophrenia. See Table 1 for a summary of recommendations for diabetes management in these patients. As seen above, they are at risk not simply for diabetes but also for other metabolic conditions that lead to increased cardiovascular risk and mortality. The first step in diabetes management is prevention.

Prevention of obesity is an important part of preventing diabetes in schizophrenia patients as in the general population. Comprehensive programs to improve diet and physical activity of people with schizophrenia and other mental illnesses have been shown to be effective for clinically significant weight loss [35] as well as metabolic parameters. One program showed a greater decline in fasting blood sugars compared to controls [36]. Evidence has shown that for these programs to be effective they have to be of longer duration, include both education and activity within group settings, and include both nutrition and physical activity components. Manualized programs may be more successful than unstructured interventions. Individual clinicians can also provide office based counseling with specific targets for behavior change and periodic monitoring of weights and metabolic parameters [37]. This should happen at both the primary care site and the psychiatrist's office.

Another key strategy in preventing diabetes is periodic monitoring of patients. Most expert recommendations argue for frequent monitoring of metabolic parameters in those with schizophrenia [38]. These patients should be considered high risk regardless of age and presence of other risk factors. A targeted approach to screening should be employed in all patients, but frequency of screening will need to be higher in those on antipsychotics. In this particular patient population, adherence and follow-up may be an issue and so glycosylated hemoglobin (HbA1c) may be preferable to a fasting glucose level as a screening test. For those on antipsychotics, screening should be done at baseline and then at 3-month intervals. If the HbA1c levels remain stable, screening can be reduced to 6-month or 1-year intervals.

Primary care physicians should also confer with the treating psychiatrist when a patient with schizophrenia gains excessive weight or develops glucose intolerance. The treating psychiatrist may not be aware of the development of glucose intolerance and other metabolic abnormalities. Also, the psychiatrist may be able to change the antipsychotic medication if the patient is on an agent that has a high risk of causing obesity and diabetes. Among antipsychotic agents, ziprasidone has shown to be the least likely to cause significant weight gain and should be considered in all patients [39]. Aripiprazole and other antipsychotics such as perphenazine, fluphenazine, and haloperidol can be considered as second line agents to switch to for attenuation of weight gain. As described above, clozapine and olanzapine are the antipsychotics most likely to cause weight gain as well as hyperglycemia and other metabolic complications. Risperidone and quetiapine are next in line in terms of likelihood of causing obesity and diabetes. While every attempt should be made to switch to an antipsychotic agent with lower metabolic risks, it should be remembered that the metabolic abnormalities are not always reversible with cessation of the offending agent. Also clozapine represents a special case since its use is limited to patients who failed other antipsychotics; thus a switch out of clozapine may not be possible.

Many pharmacologic agents have been studied in patients with schizophrenia and other psychotic disorders to prevent or reverse the weight gain and metabolic abnormalities found in patients treated with antipsychotic medications [40]. Metformin is a promising agent as it has the potential for modest weight loss and improves insulin sensitivity and glucose regulation. The Diabetes Prevention Study showed that, in the general population, metformin is effective in preventing conversion to diabetes in those with impaired glucose tolerance but less effective than lifestyle interventions aimed at improving eating and physical activity behavior [41].

In people on antipsychotics, metformin has been studied for both prevention and treatment of antipsychotic-induced weight gain. It has been shown to be effective in attenuating weight gain on antipsychotics and improving glucose regulation [42, 43]. Olanzapine is the most commonly studied antipsychotic in the metformin trials. In a sample of patients with chronic schizophrenia on olanzapine, metformin was effective in reducing both weight and HbA1c levels [44]. The weight loss compared to placebo was modest at 2 kg, which is similar to weight loss observed with metformin in the general population. In another cohort of patients who were early in the course of schizophrenia, metformin was superior to lifestyle interventions but combined intervention was superior to either intervention alone [45]. Mean reduction in body mass index (BMI) was about 1.8 in the group that received both metformin and lifestyle interventions compared to an increase in BMI of 1.2 in the placebo group. The fasting glucose decreased by a mean of 7.2 mg/dl in the metformin and lifestyle interventions group while it increased by 1.2 mg/dl in the placebo group. Metformin has also shown efficacy in improving weight and metabolic profile in patients on clozapine [46].

Thus, metformin has shown efficacy in improving glucose regulation in those with schizophrenia, though the effect sizes are small as in the general population. It should be considered early in the course of illness in all patients with schizophrenia who are obese and have evidence of glucose dysregulation, even if they are not on antipsychotics. However, given that the reductions in weight and glucose are small, metformin is only one step in the treatment of those with glucose intolerance.

The bulk of the data on metabolic abnormalities and antipsychotics is on weight gain related to these agents. Besides metformin, topiramate, an anticonvulsant, has shown some success in treating antipsychotic related weight gain [47]. Some prominent side effects such as cognitive impairment may be a deterrent for its use in schizophrenia. Other available weight loss agents such as orlistat, lorcaserin, and naltrexone/bupropion combination have not been adequately studied in the schizophrenia population. None of these agents affect glucose metabolism. Thus it is not clear if they can prevent conversion to diabetes. Other novel agents used for treatment of diabetes are being studied for diabetes prevention in prediabetic patients. However, the potential benefits both in the general population and in those with schizophrenia are unknown at this time.

Diabetes care in those with schizophrenia should be intensive and started early. As in all patients, the primary goal is to prevent long-term complications. Patients with schizophrenia have multiple other cardiovascular risk factors. Other conditions such as tobacco dependence and hypertension should be aggressively treated. Obstructive sleep apnea is another cardiovascular risk factor that is highly prevalent in this population [48]. As outlined above, diet and exercise measures can be effective and patients should be referred to a structured treatment program, if available. At a minimum, they should be referred to a nutritionist for specific recommendations on dietary modifications for managing diabetes.

At all stages of prevention and treatment, providers should communicate with family members or other caregivers. In patients with schizophrenia, issues of adherence and follow-up may be even more problematic than in the general population. Due to psychiatric symptoms and cognitive deficits, longer visits and simplified explanation of recommendations may be necessary. The long-term risks of poorly treated diabetes, including increased mortality, should be clearly delineated. Providers should also recognize that symptoms such as paranoia or delusions might interfere with adherence to recommendations. For this reason, providers should arrange for frequent follow-up visits, especially in initial stages of treatment. Providers will also need to coordinate care with specialists for routine diabetes care such as visits for a fundoscopic eye exam.

Adequate hygiene may be a problem in some patients and so special attention should be paid to foot care. They may not report neuropathic symptoms readily and a foot exam should be done at every visit. In the event of vascular complications with development of a diabetic ulcer, wound care should be aggressive with close follow-up. Poor dentition is frequently seen in these patients and efforts should be made to obtain dental evaluation and treatment to prevent gingivitis and other infectious complications. Sexual dysfunction, which can result from diabetes, can also be due to antipsychotic treatment. These medications affect sexual function through multiple mechanisms including elevated prolactin levels. Similarly, gastroparesis, a complication of diabetes, can also result from anticholinergic medications that are often used to combat adverse effects of antipsychotics.

Attention should also be paid to psychotropic medications that can cause chronic kidney disease. Lithium is a mood stabilizer used in patients with schizophrenia and coexisting mood disorder. Long-term treatment with lithium is associated with a range of glomerular and tubular disorders resulting in chronic kidney disease and rarely renal failure [49]. Potential interactions between psychotropics and agents used to treat diabetes and comorbid hypertension and hyperlipidemia should also be taken into consideration [50].

It is worthwhile to note that some antipsychotic medications, especially quetiapine and olanzapine, have been associated with acute onset of hyperglycemia and ketoacidosis [51]. Cognitive dysfunction associated with elevated glucose levels may be mistaken for the patient's baseline cognitive deficit resulting in delay in detection of hyperglycemia.

Other measures to prevent and treat comorbidities, such as use of aspirin and preventive immunizations, should be based on current evidence-based guidelines, as with any diabetic patient.

Antidiabetic agents with lesser likelihood of weight gain should be used whenever possible, since these patients are already on psychotropic medications that cause weight gain. If they are on medications that can cause hypoglycemia, very specific recommendations on immediate management should be provided to both patients and caregivers. Similarly, if self-monitoring with finger stick glucose measurements is necessary, caregivers may have to be involved for those patients with significant impairments. The frequency of glucose self-monitoring should also be tailored to the patient's capabilities. Target HbA1c goals should be individualized. The cardiovascular benefits of intensive glucose control should be balanced against the risks of hypoglycemia and the capacity of the patient to self-manage this complication of treatment. Providers should be flexible in tailoring treatment goals to each individual patient.

If aggressive lifestyle interventions and pharmacotherapy are inadequate to achieve target glucose levels and insulin administration becomes necessary, the insulin regimen should be kept as simple as possible. A basal insulin regimen offers the benefit of lesser risk of hypoglycemia. But in those whose level of hyperglycemia warrants additional prandial insulin, a premixed insulin regimen may be simpler to administer. Tight glucose control may not be possible in all patients. For any insulin regimen, patients should receive specialized nursing education and this may have to be delivered over multiple sessions. Home nursing services can be arranged in areas where such resources are available. Family members and caretakers can be trained in supervising or administering finger-stick glucose measurements and insulin injections. Depending on the practice setting, the treating psychiatrist may be able to assist with arranging for specialized services in the community, such as additional case management or nursing services. Coordination of care with the psychiatrist is of paramount importance.

Finally, bariatric surgery should be considered in patients with severe obesity with or without diabetes. It has been shown to improve diabetes measures beyond what is expected with weight loss in the general population [52]. Studies of bariatric surgery in patients with schizophrenia are lacking. However, bariatric surgery is effective in patients with bipolar disorder and psychiatric outcomes are not worse [53, 54]. Therefore, patients with serious mental illness, if selected and managed appropriately, can be good candidates for bariatric surgery. Patients with schizophrenia should not be denied the procedure solely on account of their mental illness. In those who have failed other measures, it may be the only treatment option, especially in severely obese patients with coexisting diabetes.

## 5. Conclusion

Patients with schizophrenia represent a high-risk population for developing diabetes. The etiology is multifactorial and is a combination of genetic susceptibility, common biologic pathways, environmental factors, and treatment with antipsychotic medications. In spite of increased awareness of the high cardiovascular morbidity and early mortality in this population, rates of screening and treatment remain low. These patients often do not engage adequately in treatment with their primary care providers. An appropriate treatment milieu should be provided to better engage patients in treatment. A key strategy in preventing and treating diabetes and other components of metabolic syndrome should be prevention. Patients with schizophrenia should be seen and evaluated periodically to screen for diabetes and other cardiovascular risk factors. Aggressive lifestyle interventions should be employed for those with obesity and prediabetes or diabetes. Metformin has potential for attenuating weight gain and preventing diabetes and should be considered early in at-risk patients. Pharmacologic measures and bariatric surgery can be as effective in the schizophrenia population as in the general population. However, psychiatric symptoms or cognitive deficits often interfere with optimal diabetes care in these patients and primary care providers should pay special attention to their individualized needs. Providers should collaborate with treating psychiatrists to optimize both medical and psychiatric treatment to prevent the early mortality seen in this vulnerable population.

## Figures and Tables

**Table 1:** Special considerations for diabetes treatment in schizophrenia patients.

Prevention	Refer patient to structured program for weight management as lifestyle interventions are proven to work.

Screening	Perform screening frequently: every 3 months when staring antipsychotic, then every 6–12 months. HbA1c is preferable to fasting blood sugar.

Medication switch	Confer with psychiatrist to change to medication with lower weight gain potential, if clinically feasible.

Patient education	Provide simplified recommendations as cognitive impairment may limit learning. Schedule longer medical visits.

Treatment adherence	Arrange frequent follow-ups as compliance is often poor. Communicate treatment plan to family and caregivers.

Diabetes care	Tailor frequency of glucose self-monitoring to patient capability. Arrange home nursing services if available. Address foot care at each visit as hygiene is often poor. Treat wounds aggressively if skin breakdown is present. Refer patient to dental care to prevent gingivitis.

Psychotropic side effects that mimic or exacerbate diabetes symptoms and complications	Psychotropic side effects include gastric slowing from anticholinergic agents, sexual dysfunction from antipsychotics and antidepressants, and chronic kidney disease from lithium.

Pharmacologic treatment	Set flexible target HbA1c goals as hypoglycemia from tight glucose control may be difficult to self-manage. Consider metformin early as proven efficacious in this population. Prescribe basal or premixed insulin for easier management.

Surgical management	Refer patient to bariatric surgery if eligible by weight criteria.

Comorbid illnesses	Screen for and treat high prevalence conditions like tobacco dependence, obstructive sleep apnea, and hypertension.
